# Protein Adsorption: A Feasible Method for Nanoparticle Functionalization?

**DOI:** 10.3390/ma12121991

**Published:** 2019-06-21

**Authors:** Roberta Cagliani, Francesca Gatto, Giuseppe Bardi

**Affiliations:** 1 Nanobiointeractions & Nanodiagnostics, Istituto Italiano di Tecnologia, Via Morego 30, 16163 Genova, Italy; roberta.cagliani@iit.it; 2 Department of Chemistry and Industrial Chemistry, University of Genova, Via Dodecaneso 31, 16146 Genova, Italy; 3 Drug Discovery and Development Department, Istituto Italiano di Tecnologia, Via Morego, 30, 16163 Genova, Italy; francesca.gatto@iit.it

**Keywords:** nanoparticles, protein corona, drug delivery, surface functionalization

## Abstract

Nanomaterials are now well-established components of many sectors of science and technology. Their sizes, structures, and chemical properties allow for the exploration of a vast range of potential applications and novel approaches in basic research. Biomedical applications, such as drug or gene delivery, often require the release of nanoparticles into the bloodstream, which is populated by blood cells and a plethora of small peptides, proteins, sugars, lipids, and complexes of all these molecules. Generally, in biological fluids, a nanoparticle’s surface is covered by different biomolecules, which regulate the interactions of nanoparticles with tissues and, eventually, their fate. The adsorption of molecules onto the nanomaterial is described as “corona” formation. Every blood particulate component can contribute to the creation of the corona, although small proteins represent the majority of the adsorbed chemical moieties. The precise rules of surface-protein adsorption remain unknown, although the surface charge and topography of the nanoparticle seem to discriminate the different coronas. We will describe examples of adsorption of specific biomolecules onto nanoparticles as one of the methods for natural surface functionalization, and highlight advantages and limitations. Our critical review of these topics may help to design appropriate nanomaterials for specific drug delivery.

## 1. Introduction

Nanoparticles (NPs) released in biological fluids are immediately covered with biomolecules that change their size and physicochemical properties [1]. NPs usually adsorb these molecules onto the external side, forming what is described as a “corona”. The corona includes proteins and possibly extracellular matrix components depending on the size and composition of the biological milieu. Many different physical events contribute to a corona’s formation, such as temperature, pH, and the time of interaction between the NPs and the medium’s components.

A protein corona is actually made up of two distinct adsorbed layers: the “hard corona” and the “soft corona” [2]. The hard corona contains proteins with a higher affinity for the NP surface that may irreversibly bind the chemical moieties on the particle. In contrast, the soft corona layer harbors proteins that have a lower affinity for the NP external chemistry and often adhere with reversible interactions. The exposure time is crucial for this dynamic process, as it increases the chances to stabilize the interactions and regulates, in part, the type and number of bound molecules.

Many of these proteins increase NP internalization into cells by specific binding with their cognate receptors expressed on cell membranes [3], whereas others are reported to impede NP internalization, such as CD47 or clusterin [4,5]. These observations have encouraged some researchers to use these mechanisms as a strategy to exploit the protein corona for NP surface functionalization aimed at type-specific cell delivery [6]. However, precise ligand–receptor interactions imply the preservation of the native structures of the adsorbed proteins. Their function can be compromised by protein denaturation, steric hindrance (crowding), an unfavorable orientation due to the rearrangement of their spatial configurations to adapt to the NP surface, and, ultimately, unspecific clustering of the other components of the corona [7].

In the present review, we discuss the approach to creating specific protein coronas as a sort of “natural nanomaterial functionalization”, allowing for increased or decreased interactions with cells. Although encouraging results have been obtained with this method, many structural limitations, immune responses, and species-specific differences give rise to concerns about the successful application of this strategy in future clinical NP-mediated drug delivery.

## 2. Protein-Adsorption-Mediated Targeting of Nanoparticles

The physiological behavior and fate of the NPs are dictated by the corona components that change the original properties of the particles. Protein coronas confer a “biological identity” on the NPs [8], being what cell membranes really sense. The adsorbed molecular composition represents a challenge for feasible medical applications of NPs, as it impacts on the biodistribution of nanotherapeutics and modulates their efficacy [9].

Intravenously administered NPs undergo the action of the mononuclear phagocyte system (MPS) that recognizes foreign material after being marked by the adsorption of specific serum proteins in a process called opsonization. If opsonins (blood proteins, such as immunoglobulins (Ig) and complement factors) are present in the corona, they promote the cellular uptake of NPs through the opsonin-cognate receptors expressed on the phagocyte surface [10,11]. In addition, the protein corona could conceal specific ligands in the NP surface, influencing the targeting capability. Studies have found that this ability can be lost or retained in the presence of plasma proteins [12,13].

### 2.1. Coronas and the Prevention of Cell Recognition

For many years, the main adopted strategies aimed to reduce protein adsorption to prevent immunological recognition and preserve ligand exposure. For instance, hydrophilic polymers are often grafted onto the surface of NPs in order to reduce interactions with plasma components. Currently, the most used polymer is Polyethylene glycol (PEG) due to its near-neutral and hydrophilic properties [14,15]. However, this functionalization does not completely suppress protein binding [16,17]. Actually, some results indicate that the PEG “stealth” effect could be due to the creation of a specific protein corona. Schöttler and colleagues studied human plasma protein adsorption onto PEG and poly(ethyl ethylene phosphate)-modified polystyrene NPs. The presence of polymer chains on the NP surface did not prevent protein adsorption, but changed the corona’s composition, recruiting specific apolipoproteins. Among them, ApoJ (also called Clusterin) plays a predominant role as a dysopsonin, namely, it reduces non-specific cellular uptake in murine macrophages [5]. Although other unknown molecules contribute to this phenomenon, Clusterin has been shown to also prevent the internalization of non-PEGylated NPs, including silver and silica NPs [18]. Therefore, immune cell escape does not seem to be a feature of the polymer shell per se, but it does require specific protein binding.

Many studies have demonstrated that pre-formed protein coronas of other dysopsonins can control NP–cell interactions. One of them is albumin, the most abundant protein in the blood, that has been used to develop a protective coating that avoids plasma protein adsorption and prolongs the NP circulation time. It is worth mentioning that albumin is and has been frequently identified in the protein corona of several types of NPs, modulating their tissue localization and cell targeting. Several studies on albumin-coated NPs made of different bulk materials can be found in the literature. For example, in the study of Ogawara et al., the authors observed that pre-coating polystyrene nanospheres with human serum albumin (HSA) decreased NP association with blood components and reduced hepatic targeting of intravenously injected nanospheres [19]. The same group also investigated the effect of an albumin coating on the in vivo disposition of PEGylated liposomes after intravenous administration in rats. The results showed a prolonged blood circulation time due to a low amount of associated proteins [20]. Surprisingly, this strategy seemed to be efficient regardless of the species-specificity. In fact, Peng and co-workers developed Poly-3-hydroxybutyrate-co-3-hydroxyhexanoate (PHBHHx) NPs with a bovine serum albumin (BSA) corona. They found that the pre-formed corona reduced the subsequent adsorption of IgG and the complement fragment C4b on the NP–BSA surface, ultimately resulting in lower opsonization after serum exposure in rats. The pre-formed BSA corona behaved as a protective coating in vivo, reducing the clearance speed and extending the NP circulation time [21].

Conversely, it has been shown that albumin pre-coated NP interactions often depend on the chosen experimental model. Nguyen and colleagues demonstrated cell-specific differences in the uptake of gelatin-oleic NPs with an albumin pre-coating. In this case, the administration of BSA corona–NP complexes resulted in decreased A549 cell uptake with or without fetal bovine serum (FBS) in the medium. In contrast, a strong increase in cellular uptake by HEK 293 cells has been observed [22].

### 2.2. Coronas as a Targeting Tool

Along with the mentioned exploitation of dysopsonin pre-coating of NPs, an emerging strategy for a targeting purpose relies on an “ad-hoc-designed” nanomaterial chemical surface with the ability to control the corona’s formation. NPs are synthesized to promote interactions with specific plasma components that naturally target specific cells. This selective protein adsorption actively drives NPs to the desired cellular destination [6].

One of the first examples was the use of polysorbate-80 (also known as Tween 80), a nonionic surfactant derived from polyethoxylated sorbitan and oleic acid. A polysorbate-80 NP shell promotes the adsorption of apolipoprotein subsets (mainly ApoE) that allow for the transport of surfactant-coated NPs across the blood–brain barrier (BBB) to bind the low-density lipoprotein (LDL) receptor [23,24,25]. Brain targeting through the same mechanism has been also shown for Polybutyl cyanoacrylate (PBC) and polymethyl methacrylate (PMMA) NPs covered by polysorbate [26,27,28]. Likewise, other surfactants (e.g., poloxamer) have been used to transport the antitumor drug doxorubicin across the BBB [29,30]. Apolipoproteins’ role as specific targeting molecules was also investigated by Kim and collaborators. This study showed that poly(ethylene glycol) poly-hexadecylcyanoacrylate (PEG-PHDCA) NPs preferentially absorbed rat ApoE and ApoB-100 on their surface and were effectively taken up by rat brain endothelial cells using the LDL receptor on the BBB [31]. However, a major problem regarding the off-targets of these delivery strategies must be considered. Indeed, in addition to the brain, LDL receptors are expressed on liver hepatocytes [32], leading to possible toxic outcomes and challenging the specificity of the target tissue.

Besides apolipoproteins, diverse molecules with targeting specificity have been studied. Caracciolo and colleagues used 1,2-dioleoyl-3-trimethylammonium propane (DOTAP)/DNA cationic liposome/DNA complexes (lipoplexes) to deliver nonviral nucleic acid into tumor cells [6]. The lipoplexes were pre-incubated in human plasma to form a protein corona with prevalent adsorption of vitronectin and albumin on its surface. Vitronectin contains the arginine-glycine-aspartic acid (RGD) motif and was chosen as a promising targeting protein to recognize α_V_β_3_ integrins, which are overexpressed in many solid tumors. The results showed increased internalization of NPs to vitronectin-receptor-positive cells and reduced internalization in cells with a lower expression of α_V_β_3_ integrins.

Actually, liposomes’ specific avidity for plasma proteins containing the RGD motif has clinical relevance due to their preferential targeting of pancreatic cancer cells [33]. Palchetti and colleagues analyzed the protein corona fingerprints of 10 different liposomal formulations and showed that the predicted targeting capability of the corona-coated NPs is correlated with the cellular uptake in pancreatic adenocarcinoma (PANC-1) and insulinoma (INS-1) cells [34].

Zhang et al. demonstrated the preferential recruitment of a native transport protein (retinol binding protein 4, RBP) on the surface of retinol-conjugated NPs. This specific protein corona successfully directed the coronated NPs into hepatic stellate cells (HSCs). Since HSCs act as fibrogenic cells in hepatic fibrosis, the formation of retinol–RBP complexes creates a potential efficient nanocarrier for antifibrogenic drug release [35].

A similar strategy was followed by Santi and co-workers using the spontaneous recruiting of transferrin (Tf) by gold NPs conjugated with a specifically designed peptide [36]. The Tf-binding peptide efficiently interacted with Tf, at the same time showing low non-specific adsorption. Peptide-functionalized NPs enhanced the internalization by Tf-receptor-positive cells, including in the presence of human plasma containing a physiological level of Tf.

Antibody pre-coating of NPs has also been investigated. Tonigold et al. demonstrated that the pre-adsorption of antibodies against the CD63 antigen of monocyte-derived dendritic cells or the T lymphocyte CD3 antigen exerts remarkable targeting properties, proving that pre-coating with particular antibodies could be a promising approach to targeted NP-mediated delivery. Interestingly, noncovalent targeting ligands have shown a better targeting efficiency than covalent ligands [37]. These data are supported by observations of Simon et al. showing that IgG-depleted human plasma creates a protein corona on polystyrene NPs that prevents interactions with rat macrophages, also demonstrating the stability of the pre-formed corona in the presence of complete plasma [38]. Conversely, Mirshafiee and colleagues engineered NPs to promote the adsorption of immunoglobulins with the aim of enhancing NP uptake by macrophages. The authors precoated silica NPs with human gamma immunoglobulins to produce an opsonin-enriched corona. They found no enhancement of internalization by murine macrophages [39].

**Figure 1:**
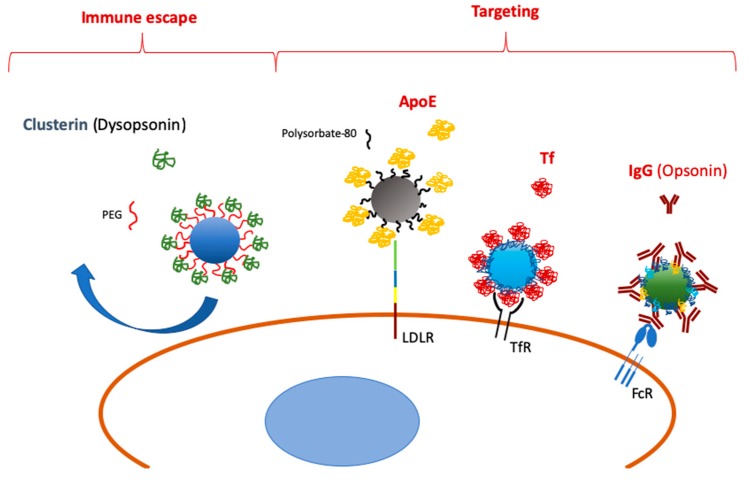
Scheme of selective protein adsorption onto nanoparticles (NPs) leading to immune escape and specific targeting.

Selective recruitment of adsorbed molecules onto NP surfaces is an emerging option and an intriguing way to drive the fate of NPs used for drug delivery (Figure 1). Particularly, pre-coating with antibodies could be advantageous considering their size and structural stability in the blood of these proteins. As we will see in the next paragraphs, however, some limitations, such as protein corona conformational changes and the predominant defensive role of complement- and Ig-mediated opsonization, should be considered before enrolling this method for translational medicine purposes. Furthermore, a species-specific mismatch (i.e., human plasma vs. murine immune cells) could produce controversial results due to unexpected interactions between ligands and cognate receptors of different species.

## 3. Limitations of Protein-Adsorption-Mediated Targeting

### 3.1. NP-Dependent Protein Modifications

Almost a quarter of a century ago, Borchard and Kreuter described the role of non-complement plasma components in the phagocytosis of injected NPs in vivo [40]. Remarkably, they found that the plasma protein corona was able to avoid uptake by the rat MPS, especially after heat inactivation. Different mechanisms induced modifications in the coating’s proteins that impaired cellular internalization of NPs, including temperature-dependent structural changes.

The interaction between corona proteins and NP surfaces may lead to reversible or irreversible rearrangements of the peptides. These alterations are regulated by the physicochemical surface features and the surrounding environment [41]. Although minimal changes could be regained following detachment from the NP, significant modifications, such as loss of *β*-sheets or *α*-helixes, cannot be restored and compromise the protein function. As mentioned above, these effects depend on a variety of factors, including NP surface chemistry, size, and shape, as well as protein sequence, conformation, and hydrophobicity. The precise detection of structural modifications occurring in adsorbed proteins is very challenging and requires the coordinated employment of several techniques, including Dynamic Light Scattering (DLS), electron microscopy, electrophoresis, Circular Dichroism (CD), Fourier Transform Infrared Spectroscopy (FTIR), Mass Spectroscopy (MS), and Nuclear Magnetic Resonance (NMR) [42]. These techniques are summarized in Table 1.

The ratio between NP and protein size is an important factor determining the amount of adsorbed proteins and alterations in their structure. It is realistic to presume that if the NP is much bigger than a single protein, more peptides can be harbored on the NP surface. Besides, a low curvature of the NP surface may induce globular protein stretching to adhere to a quasi-flat floor. On the other hand, smaller NPs of the same material have less contact with protein domains and a lower chance to induce structural variations [41]. In agreement with this hypothesis, 110 nm citrate and Polyvinylpyrrolidone (PVP)-stabilized AgNPs were found to bind a higher number of proteins compared to 20 nm citrate and PVP-stabilized AgNPs, suggesting a different corona formation due to size and surface curvature of the NPsn [43]. Moreover, as reported by Kurylowicz et al., the interaction between two different proteins was reduced on highly curved polystyrene NP surfaces compared to flat polystyrene nanofilms [44]. Nevertheless, Dobrovolskaia and colleagues observed by two-dimensional (2D) PAGE that 30 nm colloidal citrate–gold NPs incubated in human plasma bind more proteins than 50 nm NPs of the same composition. The authors suggest that a multilayered interaction occurs, such as a cationic protein binding the gold anionic surface at one site and another anionic protein on the other site [45]. NP size has been confirmed to have an influence on a protein corona’s composition for different nanomaterials (NMs) [10]. Although it is not always straightforward to determine whether corona proteins have undergone structural changes, non-specific aggregation of several biomolecules often leads to an unexpected folding and loss of tertiary structures. Actually, it has been shown that the adsorption of certain proteins on 100 nm NPs induces increased protein modification compared to particles of the same material smaller than 5 nm [46].

Along with the relevance of NP size, it is worth highlighting the key role of NP shape and surface morphology in protein corona formation. For instance, titanium dioxide NPs of a spherical shape bind proteins that are not found on rod-like particles [47]. As shown by Scopelliti et al., non-homogeneous adsorption creates clusters close to pores and grooves on the surface of mesoporous NPs. In addition, their results show that the number of nucleation sites increases as the surface roughness increases [48].

As expected, NP surface chemical moieties and charge can dramatically influence protein adsorption and interactions with cell membrane components [46,49,50]. Differences in the complexity and abundance of corona biomolecules can also be related to the free energy in protein folding and unfolding induced by different surface groups of the same size [43]. Modification of NP polymeric composition and polymer size promotes structural changes in adsorbed proteins, as shown for the loss of α-helixes in adsorbed albumin [51]. Similarly, large PEG chains placed on the NP surface can inhibit the formation of a BSA corona [52].

NPs with neutral shells usually bind less proteins than NPs with negatively or positively charged surfaces [53]. Studies on polystyrene NPs demonstrated that proteins with an isoelectric point of less than 5.5 (e.g., albumin) mainly adsorbed on positively charged particles, whereas proteins with higher values (e.g., IgG) prefer negatively charged NPs [54]. So, although they are nonprotein-corona-specific, NP functionalization with negative (e.g., COOH) or positive (e.g., NH_2_) chemical groups may represent a way to select the protein coating.

In a detailed spectroscopic study, Podila and colleagues evaluated the interaction between BSA and carbon nanostructures, such as graphene and single-walled carbon nanotubes (SWNTs). Charge transfer to BSA occurred in the presence of SWNTs but not in the presence of graphene. This effect may be due to the sharp and discrete electronic density of states of SWNTs. The increase in charge transfer corresponded to the relaxation of external α-helices in the BSA’s secondary structure [55].

Further modifications of corona proteins in response to several environmental factors, including pH, temperature, salt composition, and protein and particle hydrophobicity, have been appropriately and carefully reviewed elsewhere [41,46].

### 3.2. Predominant Role of Complement and Antibody Opsonization

As mentioned above, NPs released in the blood stream quickly interact with several proteins of diverse molecular weight, aminoacidic composition, and cellular origin [56,57]. Opsonization by the complement system’s molecules and antibodies is a common event shared by all intravenously injected NMs [58]. Recently, important data regarding protein adsorption onto NPs were collected by Chen and colleagues using dextran-coated superparamagnetic iron oxide (SPIO) nanoworms incubated in human serum and plasma [59]. Rapid opsonization of the nanoworms with C3 via an alternative pathway was observed. The authors demonstrated that C3 was covalently bound to the adsorbed proteins inserted into the dextran shell. C3 opsonization was also shown to be a reversible and dynamic process, suggesting its binding to the soft-corona-forming proteins. A critical role in this process is played by immunoglobulins [60]. Regardless of the activation pathway, natural antibodies bound to the adsorbed proteins on the NP surface trigger complement activation. Vu et al. propose two potential mechanisms of C3 activation and the binding of its proteolytic component C3b: (1) protein corona binding by immunoglobulins, which in turn are attacked by spontaneously formed C3b; and (2) the previous formation of an IgG–C3b complex followed by the binding of the immunoglobulin moiety to the proteins, which are adsorbed onto the NP surface. Different human complement molecules, such as C1q, have also been shown to directly bind with high affinity to a Poly(2-methyl-2-oxazoline) (PMOXA)-coated silica NP’s surface and enhance C3 opsonization of the particle [61]. Antibody and complement proteins that bind to NPs accelerate their recognition by immune cells and, in particular, their capture by phagocytes. Interestingly, the same PMOXA-coated NPs injected in vivo were not able to activate the mouse complement system and were poorly internalized by mouse macrophages. This observation poses a crucial question about the experimental setup and the reliability of the data collected from different biological models. We have already discussed and revised the general significance of an NP surface coating and the experimental models in vitro and in vivo [62,63] to study the immune compatibility of NMs. The different affinities of a particular surface material for species-specific complement molecules highlights the complexity of immune system interactions with NMs and their validation for biomedical applications.

The avoidance of complement-mediated phagocyte sequestration of NPs remains one of the major issues in nanotechnology applications to drug delivery [64]. Poly(ethylene glycol) (PEG) covering of NM surfaces is a well-established method to delay phagocyte sequestration and allow for a prolonged NM or therapeutic presence in the blood circulation by increasing their hydrodynamic radius and retarding protein adsorption [32]. As described in the previous paragraph, a potential mechanism by which the presence of PEG limits NP cellular internalization has been partially described by Schöttler et al. [5]. PEG interaction with specific plasma proteins, such as clusterin, has been shown to be an essential event to avoid cellular uptake. PEGylated NPs, previously incubated with human plasma proteins, have been added to adherent murine RAW 264.7 macrophages in serum-free medium. Although the authors’ flow cytometry and confocal microscopy results support the proposed mechanism, the experimental setup did not represent a realistic model. Human protein–corona interactions with an adherent murine cell receptor do not provide information on the intra-species interface events that regulate PEG–NP contacts with circulating immune cells. In contrast to Schöttler et al., Tavano and colleagues [61] found no significant correlation between the presence of clusterin on the NP surface and recognition by macrophages. Furthermore, they describe the role of clusterin in the regulation of complement activation as one that accelerates the formation of one of its components, namely sC5b-9. Anti-PEG IgG and IgE production and their role in the development of immediate hypersensitivity [65] or complement-activation-related pseudo-allergy (CARPA) [66] suggest that caution be exercised in the clinical use of PEGylated NMs.

## 4. Conclusions

All of the presented observations demonstrate the significance of unraveling nano–bio interactions with immune system components to improve the drug delivery systems and their targeting efficiency. Preparing NPs with a composition and chemical surfaces that allow for the adsorption of stable molecules to create a protein corona with a specific target could be very encouraging for several clinical treatments. In addition to antibodies, different proteins resident in blood could theoretically be used to create coronas with the ability to modulate NP localization on purpose. Molecule stability on NP surfaces in vivo is one of the most serious concerns at present, and will require coordinated and continuous interdisciplinary research in the future.

## Figures and Tables

**Table 1:** The main techniques used to study protein corona features.

Analytical Technique	Detection	Characterization of Protein Corona
Dynamic light scattering (DLS)	Size distribution profile of small particles in suspension	NP diameter variation after the formation of protein corona
Transmission electron microscopy (TEM)	High resolution imaging	NP and protein corona imaging
SDS-PAGE (Electrophoresis)	Protein separation by mass	Evaluation of proteins’ identity in the corona composition
UV Circular Dichroism (CD)	UV spectral signature of optically active molecules	Evaluation of protein conformational changes
Fourier transformer infrared spectroscopy (FTIR)	Infrared high-spectral-resolution	Evaluation of protein aggregation and conformational changes
Mass spectroscopy (MS)	Mass-to-charge ratio of ions	Identification of corona proteins by elemental or isotopic signature
Differential scanning calorimetry (DSC)	Heat capacity over a range of temperatures	Evaluation of protein stability
Raman spectroscopy (RS)	Monochromatic light interaction with molecular vibrations	Protein–NP complex formation
Nuclear magnetic resonance (NMR)	Magnetic properties of atomic nuclei	Protein structure
